# Optic Nerve Structural and Functional Changes in LHON-Affected and Asymptomatic Maternal Relatives: Association with H and HV Mitochondrial Haplogroups

**DOI:** 10.3390/ijms24021068

**Published:** 2023-01-05

**Authors:** Clare Quigley, Kirk A. J. Stephenson, Paul Kenna, Lorraine Cassidy

**Affiliations:** Royal Victoria Eye and Ear Hospital, D02 XK51 Dublin, Ireland

**Keywords:** LHON, mitochondrial disorder, mitochondrial haplogroup, optical coherence tomography

## Abstract

Leber Hereditary Optic Neuropathy (LHON) affects a minority of carriers of causative mitochondrial DNA mutations. We investigated a cohort of patients with LHON, including m.11778G>A, m.3460G>A, m.14484T>C and *DNAJC30* c.152A>G variants, and their asymptomatic maternal carrier relatives for additional potential associations with vision loss. We assessed visual acuity, optical coherence tomography (OCT) of the peripapillary retinal nerve fibre layer (RNFL), visually evoked potential including P-100 latency, and full mitochondrial genome sequencing. Comparison was made with a reference standard for OCT; European Descent, Heidelberg Engineering ©; and electrophysiology measurements with in-house normative ranges. RNFL was thinned overall in LHON patients (n = 12); median global RNFL −54 μm in the right eye (RE) and −50 μm in the left eye (LE) versus normal, and was found to be normal overall in asymptomatic carriers at +1 μm RE and −2 μm LE (n = 16). In four asymptomatic carriers there was RNFL thinning found either unilaterally or bilaterally; these cases were associated with isolated delay in P-100 latency (25%), delay and reduced visual acuity (50%), or reduced visual acuity without P-100 latency delay (25%). Optic nerve dysfunction was associated with mitochondrial haplogroup H and HV, versus non-H haplogroups, in the asymptomatic carriers (Fisher’s exact test, *p* = 0.05). Our findings suggest that optic nerve abnormalities may be identified in asymptomatic LHON mitochondrial mutation carriers, which may be associated with optic nerve dysfunction. For asymptomatic carriers these findings were associated with mitochondrial haplogroup H and HV.

## 1. Introduction

Leber Hereditary Optic Neuropathy (LHON), first described by Leber in 1871, is an inherited optic neuropathy that usually causes blindness [1]. LHON is caused by mitochondrial dysfunction, with over 95% of LHON pedigrees associated with one of three mitochondrial DNA (mtDNA) mutations, including mt. 3460G>A, 11778G>A, and 14484T>C [2], which affect complex I (NADH-ubiquinone oxidoreductase) of the mitochondrial respiratory chain [3]. A number of other rare LHON-causing mitochondrial DNA mutations have been described [4,5,6,7,8,9,10]. Interestingly, the carrier rate for homoplasmic pathogenic mtDNA variants in the normal population is high yet the incidence of new LHON-affected cases is 1:30,000–50,000 [11].

Important unknowns in LHON include the cause of sex bias and variable penetrance in disease expression. Male sex is a risk factor for vision loss; approximately 50% of males and 10% of females with a LHON-implicated mtDNA variant go on to manifest the classic phenotype of acute (simultaneous or sequential) bilateral vision loss from optic neuropathy [12]. The protective mechanism for female sex is unknown, but speculated to be oestrogen related [13]. Other established risk factors include smoking [14] and excess alcohol [15]. In asymptomatic individuals who carry LHON-causing mutations, abnormal findings short of a clinical diagnosis of LHON may be found, including decline of PERG amplitude [16].

Mitochondrial haplogroups (groups of mtDNA single nucleotide polymorphisms (SNPs) which are inherited en masse from a common maternal ancestor) may have a role in penetrance of LHON. LHON penetrance and blindness in 11778G>A and 14484T>C in Europe has been associated with haplogroup J [17,18,19].

We prospectively investigated patients affected by LHON and their asymptomatic maternal relatives for associations of penetrance of vision loss, including demographic variables, smoking and alcohol, along with assessment of mitochondrial causative mutation, heteroplasmy and haplogroup. We also evaluated response to Idebenone.

## 2. Results

### 2.1. Demographics and Genetic Analysis

In the LHON group there were 12 subjects (3 females) from 10 pedigrees with median age 27 years, interquartile range (IQR) 19–33 years. Disease onset was at median 19, IQR 16–23.5 years. In the unaffected carrier group, comprising mothers and siblings including relatives where the LHON-affected relative was invited but unable to attend, there were 16 subjects (15 female) from 13 pedigrees, median age 56, IQR 42–61 years. In total, genetic sequencing revealed mt.11778G>A in 22 subjects, 3460G>A in 2 subjects, 14484T>C in 2 subjects, and *DNAJC30* c.152A>G homozygosity in 1 subject, see Table 1.

### 2.2. Visual Acuity Data and Optic Nerve Head Structural Findings

In the LHON group the median visual acuity in the better sighted eye was 1.6 logMAR, IQR 1.2, 2.1 (n = 12). In this group visual acuity was preserved in the better eye on on-chart (i.e., logMAR 1.0 or better) in 3 subjects (25%). Amblyopia was reported in one subject (8%). In the asymptomatic carriers, median visual acuity was 0.0 logMAR, IQR 0, 0.1 in the right eye, and 0.05, IQR 0.0, 0.2 in the left eye. Visual acuity worse than 0.5 logMAR was found unilaterally in 3 carriers (19%), they were among five carriers who reported a history of amblyopia (31%). The overall optic nerve head retinal nerve fibre layer (RNFL) was thinned in LHON subjects by median-54 microns, IQR-23, -63 microns in the right eye, and thinned by median-50 microns, IQR-62, -32 microns, in the left eye. In LHON the nasal RNFL was the only sector that was not thinned. Optic nerve head RNFL was normal overall in asymptomatic carriers, see Table 2.

**Figure 1:**
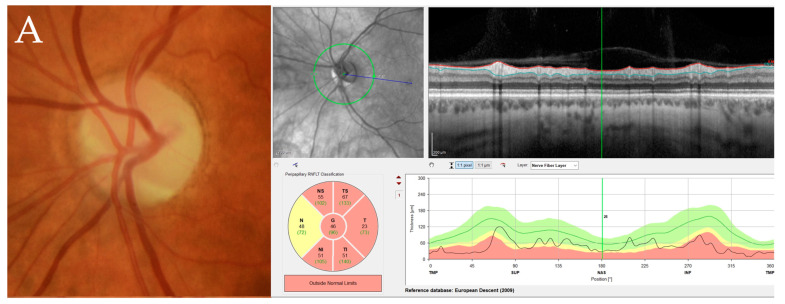

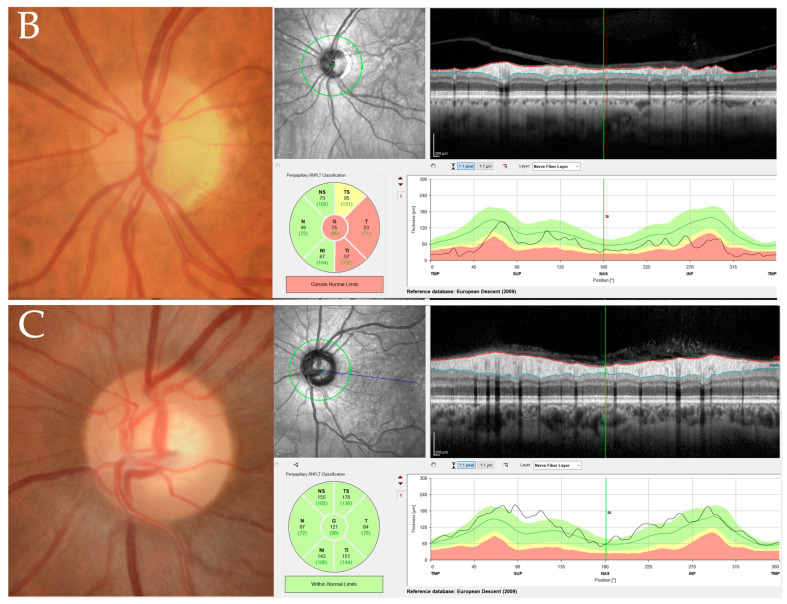
Optic nerve head photo of the left eye with corresponding optic nerve head retinal nerve fibre layer (RNFL) thickness measurement in different study subjects, C6 chronic LHON showing established optic nerve pallor (**A**), U15 asymptomatic carrier with unilateral optic nerve RNFL thinning and temporal pallor (**B**), and U5 asymptomatic carrier with normal optic nerve findings (**C**).

Due to poor fixation, assessment of optic nerve head RNFL thickness was not possible on all subjects with LHON, but was measured bilaterally in 8 subjects, and unilaterally in one subject. The RNFL was reduced in all of those with chronic LHON (n = 7), whereas thickness was normal overall, with some sectors showing thickening in early LHON (n = 2). Of asymptomatic relatives (n = 16), 75% had overall normal RNFL thickness bilaterally, 25% had reduction in RNFL in at least one eye, see Figure 1.

Of those asymptomatic relatives with reduced optic nerve head RNFL (5 eyes of 4 asymptomatic carriers), one had normal visual acuity, and the remaining three had unilateral reduced visual acuity, on the same side as the thinning in RNFL, see Table 3.

In LHON-affected patients, we tested for an association of sex with visual acuity preserved to on chart in at least one eye in LHON by Fisher’s exact test, and found no association (*p* = 0.9). We also tested for an association of sex with presence of optic neuropathy in asymptomatic carriers by Fisher’s exact test, and found no association (*p* = 0.3).

### 2.3. Optic Nerve Function, Mitochondrial Variants, Heteroplasmy, and Haplogroup

**Figure 2:**
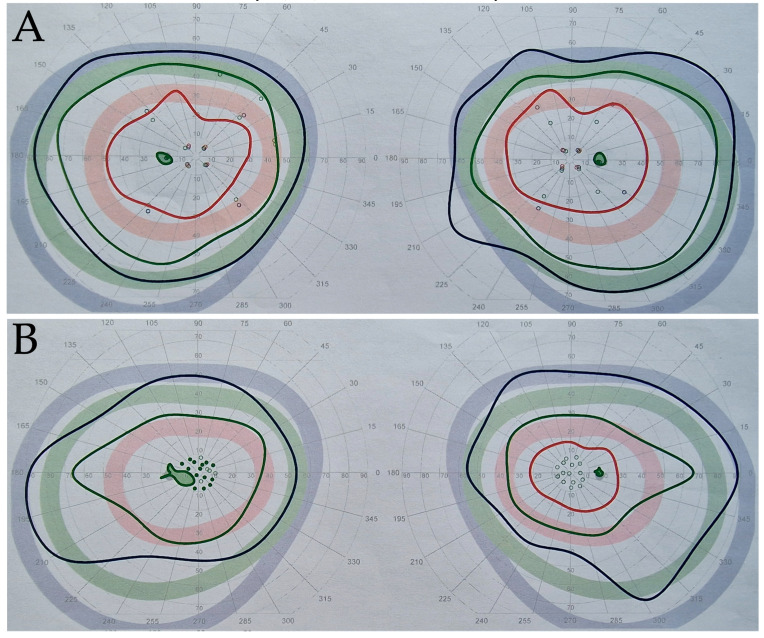

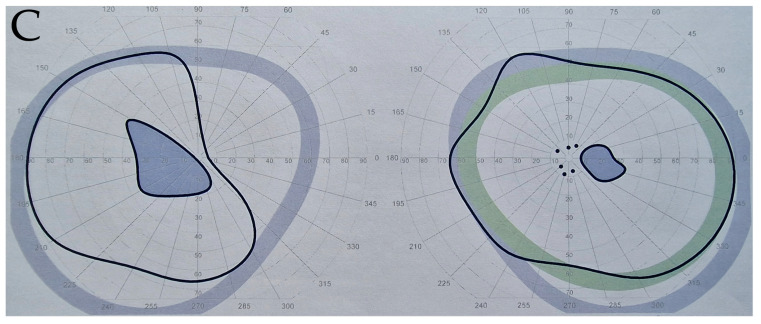
Visual field testing results showing representative classifications, with right visual field on right side and left visual field on left side of figure, I2e target in red, I4e target in green, V4e target in blue. Normal visual field bilaterally in E1 with substantially preserved I2e (**A**), mildly reduced vision field in right eye with central reduced sensitivity of I2e isopter, with moderate visual field scotomata in left eye in U15 with loss of I2e target (**B**), and bilateral severely reduced visual field with only V4e target seen in C8 (**C**).

Prolonged P100 latency was present in 100% of those affected by LHON. Visual field testing varied from normal to mild scotomata (25%), through moderate (50%), to severe visual field scotomata (25%). Carriers had normal P100 latency in 80%, with delay beyond the normal range present unilaterally in three carriers, two of whom had an abnormally high interocular difference in P100 latency greater than 10 ms, and one who had prolonged P100 latency >128 ms. Moderate visual field scotomata were present unilaterally in two of these carriers on the same side, and were found unilaterally in one other carrier, with remaining asymptomatic carriers having normal or mild visual field scotomata, see Figure 2. Of asymptomatic relatives who showed optic nerve RNFL thinning (n = 4), two of these eyes (50%) had abnormal P100 latency.

Most LHON cases were homoplasmic (83%) for a known pathogenic LHON mtDNA variant, with one case heteroplasmic (C6: 11778 G>A, 80% mutant mtDNA load in peripheral blood leukocytes), and one case where the pathogenic variant was identified to be *DNAJC30* c.152A>G (C10). In asymptomatic carriers, 69% were homoplasmic, with remaining carriers showing varying heteroplasmy (10–98%), and one carrier relative where a variant was not identified (U13, mother of C10). Analysis for association of homoplasmy ≥90% with presence of optic neuropathy in asymptomatic carriers by Fisher’s exact test was negative (*p* = 0.5). In the LHON group the most common haplogroup was HV (42%), followed by J (33%), and among the asymptomatic carriers the most common haplogroups were HV (25%), J (25%), and H (25%), for remaining haplogroups see Table 4.

In subjects with a confirmed LHON-causing variant, and electrophysiology data, mitochondrial haplogroup H or HV versus non-H was assessed for association with vision loss; in LHON-affected patients none of these were significantly associated with vision status being ‘on-chart’ (i.e., Logmar ≤ 1.0) versus ‘off-chart’, on Fisher’s exact test (*p* = 0.2), however haplogroup H or HV was significantly associated with visual dysfunction in asymptomatic carriers, on Fisher’s exact test (*p* = 0.05).

### 2.4. Idebenone Treatment

At the time of data cut-off (January 2022), all (n = 12) LHON-affected patients had received at least one dose (900 mg/day) of Idebenone and were included in the safety population (SP). Of this group, 6 patients carried one of the 3 major LHON mtDNA mutations, had onset of symptoms within the 12 months prior to starting treatment, had baseline VA data, and had at least one follow-up visit with VA data; this was the efficacy population (EP).

In the EP of 6 patients, VA was maintained at better than logMAR 1.0 in at least one eye in 2 patients (33%) while median visual acuity in the better eye was 1.2 logMAR. Of the EP group, 1 patient (C3) had a clinically relevant recovery from nadir to latest visit (17%), who had presented with visual acuity of 1.3 logMAR in both eyes and improved to 0.9 and 0.8 logMAR in the right and left eyes, respectively. The cumulative exposure to Idebenone in the SP was 214 patient months. No adverse event was reported.

## 3. Discussion

We investigated a cohort of patients affected by LHON, and also asymptomatic carrier maternal relatives, for potential associations of expression and severity of disease. We found that visual acuity was reduced to median 1.6 logMAR in the LHON group. Optic neuropathy was present in a minority of asymptomatic carrier relatives, at a rate of 25% showing signs of optic neuropathy in at least one eye, with thinning of the RNFL of the optic nerve head that was associated with reduced visual function; either reduced visual acuity or delayed visually evoked response or both. This presence of optic neuropathy in asymptomatic carrier relatives was found to be associated with membership of the mitochondrial haplogroups H or HV, versus other haplogroups, on evaluation of mitochondrial DNA. Mitochondria LHON-causing variant homoplasmy ≥90% was not associated with presence of optic neuropathy in carrier relatives, nor with severe vision loss in LHON. We found that male sex, high alcohol intake, and smoking status were not associated with worse severity of vision loss in the LHON cohort, nor with presence of optic neuropathy in asymptomatic relatives. Idebenone showed a good safety profile, with no adverse event reported over a treatment duration of 214 patient months. However, Idebenone showed limited efficacy, with 33% of those who received treatment within a year of onset of vision loss achieving clinically relevant stabilisation, with ‘on chart’ vision, and 17% achieving a clinical relevant recovery of vision.

Investigation of haplogroup membership in LHON has been previously reported in a pan-European cohort, in which membership of the H mitochondrial haplogroup was associated with relative preservation of vision [19]. Another study proposed that reduced penetrance of LHON is seen in 14484T>C in the H haplogroup, though this report included only one family [20]. Vision loss and optic nerve involvement in MS in Iran has been described in association with the H haplogroup [21]. Increased penetrance of vision loss of LHON in 11778G>A in M7b and B5 haplogroup has been suggested in Chinese studies, whereas Haplogroup F was protective [22,23]. Penetrance of vision loss and LHON expression in 11778G>A has also been described with the J haplogroup in Italy [18] and the K haplogroup in a study in Poland [24]. In these different populations different patterns of penetrance associated with mitochondrial haplogroups are observed. The mitochondrial haplogroup association also varies in our cohort in Ireland (the predominant mtDNA haplogroup in Ireland is H (40%) with significant proportions of U (12%), T (10%), J (10%) and K (10%) [25], suggesting that at a population level mitochondrial haplogroup effects may exist but that there are other important factors in LHON expression, potentially including genetic, epigenetic, and environmental exposures.

Different possibilities may explain the presence of optic neuropathy in asymptomatic carrier relatives. Most of these subjects who were found to have optic neuropathy with reduced vision report ambylopia also (75%). Amblyopia can cause prolonged P-100 latency [26], but it does not cause structural changes of the optic nerve head and does not account for these findings on OCT scanning [27]. Moreover, the prevalence of reported amblyopia in these carriers was high at 31%, compared with reported prevalence of up to 6% in international studies [28,29,30,31], indicating that there may be another underlying issue. It is possible that the carrier’s optic neuropathy is static due to impaired but not absent mitochondrial respiratory chain function causing subclinical retinal ganglion cell impairment, which has not reached the critical ATP-deprivation stage causing profound and/or bilateral vision loss. Or these subjects may represent an atypical LHON at an early stage where one eye is involved, that has not yet progressed to involve both eyes, but may go on to do so. A report has previously described a 34-year interval between losing vision first in one eye and then the other, though 97% of 2nd eye involvement in LHON occurs within 1 year [32,33]. Asymptomatic carrier relatives who show signs of LHON pose a diagnostic and management dilemma for clinicians. They may go on to manifest symptomatic optic neuropathy (i.e., pre-symptomatic group), or they may never progress to symptomatic ‘affected’ status. Distinguishing those who will progress from those who will not is not possible, thus population screening for LHON-causing mtDNA variants is neither actionable nor practical. LHON, though typically starting in early adulthood, may occur later, and onset has been described in the seventh decade of life, though 95% occur at < 50 years [33,34]. Should relatives who show optic nerve abnormalities be treated with Idebenone, with preventative or curative intent? This is unclear. Idebenone or gene therapy are the treatments for LHON, aside from avoiding optic nerve toxins including smoking and alcohol. A clinically relevant recovery of vision with Idebenone treatment has been observed in 46–53% [35,36] of patients treated in two different trials; however real world data often shows lower efficacy [37], and treatment does not prevent vision loss in the second eye [38]. Our approach is to advise these asymptomatic carriers to avoid excess smoking and alcohol.

In this cohort 75% of LHON-affected individuals carried the pathogenic mitochondrial variant 11778 G>A, with other mitochondrial variants 14484T>C and 3460G>A each seen in two other individuals. We identified the causative mutation of LHON to be *DNAJC30* c.152A>G in one affected individual from Ukraine (C10). This variant has been identified in cases of autosomal recessive LHON [10,39,40]. The DNAJC30 protein is known to associate with respiratory chain complex I [41]. Identification of this causative variant, versus the more common mitochondrial variants, impacts on genetic counselling for the affected family.

Analysis of the peripapillary RNFL in LHON-affected and carrier relatives was carried out utilising the Heidelberg European Descent, which was useful in selecting out patterns of optic nerve structural changes, in the absence of a control sample. This means of optic nerve head OCT analysis has been described recently in analysis of optic nerve head changes in compressive versus glaucomatous optic neuropathy [42]. The patterns of optic nerve changes we observed included differences in RNFL thickness associated with LHON chronicity, with preserved or thickened nerve fibre layer present in those within the first few months of their LHON onset, versus thinned RNFL in more long-standing disease. In LHON we found that the optic nerve head nasal sector had a relatively preserved RNFL overall. This may be because the temporal optic nerve head tends to be affected first [43,44], and the subjects we included were relatively young, with a median age of 27 years. Analysis of RNFL thickness in carriers yielded more information. Thinning of the peripapillary nerve fibre layer was present either unilaterally or bilaterally in a significant proportion of asymptomatic carriers, 25% (n = 4/16). For most of these carriers, optic neuropathy in these eyes was demonstrated on visual acuity testing. However, for one of these carriers, visual acuity testing was normal bilaterally, with optic nerve dysfunction detectable only upon visually evoked potential testing, which uncovered P-100 delay. This reveals the utility of visually exposed response testing yielding an abnormal finding in an eye with normal central visual acuity testing despite structural signs of optic nerve disease on OCT. We did not find any pathological increase in thickness of the temporal optic nerve head RNFL, as has been reported in Italy, in a large sample of asymptomatic carriers [45]. Temporal nerve head thickening may be detected in early disease as mismatch between metabolic supply and demand leads to localized oedema. Not finding temporal RNFL thickening in carriers in our study may be due to our smaller sample size.

Male sex [12], alcohol [46] and smoking [14] are well-established risk factors for penetrance of vision loss in LHON. Our lack of finding these associations as risk factors may be due to the small sample size and the design of our study. It is also possible that we would have detected these exposures as risk factors if we had exact details of alcohol and smoking exposure in the lead up to conversion to vision loss in LHON, but this data was not available.

Limitations of our study include that our cohort is a small heterogeneous sample of LHON patients and their maternal relatives, including four different mutation types, the majority of whom were born in Ireland (93%, n = 26), with two subjects from the same pedigree who were born in Ukraine (n = 2). We do not have a control sample for our study, but relied instead on drawing comparisons between LHON-affected, unaffected carriers, and the OCT optic nerve findings in the Heidelberg European Descent normative database which includes 201 healthy age-matched subjects, and for electrophysiology, an in-house normal sample normative database. Strengths of our study include report of mitochondrial haplogroup, previously not reported from an Irish centre of treatment of LHON, and our reporting of safety data for Idebenone, and the inclusion of 13 different pedigrees of LHON and their unaffected maternal relatives.

## 4. Materials and Methods

The Research Ethics Committee of the Royal Victoria Eye and Ear Hospital approved this study. Attendees with LHON in the neuro-ophthalmology clinic which is run by the only clinician in Ireland with access to Idebenone prescribing (LC) were invited to participate, along with their mother or another maternal relative, in a genotyping-phenotyping research study. If the patient had a sibling who was interested, they were invited to also attend. This resulted in a mixed study sample of affected patients and asymptomatic maternal relatives.

For each study participant, demographic data, including age and sex, and clinical data, including ophthalmic and medical history, medications, social and family history, were collected.

Visual acuity (VA) data was recorded, including corrected distance visual acuity via Early Treatment of Diabetic Retinopathy Study (ETDRS) charts with logarithm of the minimal angle of resolution (logMAR) values, or converted from standard Snellen notation to logMAR for analysis purposes. When BCVA was reduced to counting fingers (CF), hand movements (HM) and perception of light (PL), logMAR values were substituted as 2.10, 2.40 and 2.70, respectively [47].

Optic nerve function tests included Octopus visual field (Haag-Streit, UK) and international society for the clinical electrophysiology of vision standard visual-evoked potential and pattern electroretinogram. Dilated fundus examination was performed with colour optic nerve head photography (FF450plus, Carl Zeiss Meditec, Dublin, CA, USA), optical coherence tomography (OCT, Heidelberg Spectralis OCT © Heidelberg Engineering GmbH 2007, Heidelberg, Germany), and analysis using the SPECTRALIS^®^ Glaucoma Module, that utilizes a reference normal database of eyes of 255 normal controls, of European descent.

Participants were divided into 3 groups based on symptom presence and duration: (1) those carrying LHON mutation without symptoms or signs (asymptomatic carrier relatives), n = 16; (2) patients in the early subacute stage of disease with symptom duration <3 months (early subacute LHON, eLHON, n = 2) patients in the chronic stage of the disease >12 months (chronic LHON, chLHON, n = 10).

A peripheral blood sample was drawn to carry out genetic testing at an accredited laboratory (©Blueprint Genetics, Helsinki, Finland). Participants had genetic sequencing performed, including sequence and copy number variation analysis of the whole mitochondrial genome (37 genes), and a panel of nuclear genes including those observed to cause neuro-ophthalmic pathology (to be reported in a separate study). Mitochondrial haplogroup was derived from a reference phylogenetic tree [48], utilizing the online sequence viewing software Integrated Genomics Viewer [49].

Assessment for treatment effect of Idebenone was carried out. Clinically relevant stabilisation (CRS) was defined as baseline logMAR VA ≤1.0 in at least one eye, maintained in this eye at the latest clinic visit. A clinically relevant recovery (CRR) of BCVA was defined as an improvement from ‘off-chart’ (i.e., >1.0 logMAR), to ‘on-chart’ by at least one full line (5 letters), or an improvement in an on-chart BCVA by at least 2 lines (10 letters, 0.2 logMAR), at latest clinical visit as compared to the nadir of visual acuity during their disease course. Adverse events after commencement of Idebenone were also recorded.

## 5. Conclusions

Asymptomatic LHON carrier relatives may show signs of optic neuropathy, raising the question of the appropriate management. The presence of these signs in asymptomatic relatives is associated with membership of the H or HV mitochondrial haplogroup. In our cohort of Idebenone-treated LHON patients is well tolerated, with no adverse events, though recovery of vision was limited.

## Figures and Tables

**Table 1:** Demographic and genetic analysis data.

Characteristics		LHON-Affected	Asymptomatic Carriers
Genetic mutation	11778G>A	N = 9	N = 13
	3460G>A	N = 1	N = 1
	14484T>C	N = 1	N = 1
	*DNAJC30* c.152A>G	N = 1	
Parent country of birth: Ireland : Ukraine		N = 11 N = 1	N = 15 N = 1
Sex	Female/Male	3/9	15/1
Age (years)	Median (IQR)	27 (19–33)	56 (42–61)
Age onset		19 (16–23.5)	
		6 (2–14)	

**Table 2:** Demographic data and optic nerve head thickness: median deviation of retinal nerve fibre layer (RNFL) thickness, visual acuity, sex and age of the LHON-affected and asymptomatic maternal carrier relatives. Visual acuity is displayed in logMAR. The RNFL values are displayed in microns, and highlighted in green where normal and red where thinning is present.

Characteristics	LHON-Affected		Asymptomatic Carriers	
Female (n)	25% (3)		94% (15)	
Age (years)	27 (19, 33)		56 (42, 61)	
Median (1st quartile, 3rd quartile)				
Visual Acuity Median (1st quartile, 3rd quartile)	RE 1.8 (1.25, 2.25)	LE 1.8 (1.4, 2.1)	RE 0 (0, 0.1)	LE 0.05 (0, 0.2)
Optic nerve fibre layer (RNFL)				
Overall RNFL	−54 (−62, −23)	−50 (−62, −32)	0.5 (−9, 13)	−2 (−16, 13)
Temporal-RNFL	−48 (−38, −48)	−46 (−50, −45)	−2 (−9, 6)	−4 (−19, 4)
TS-RNFL	−83 (−97, −25)	−77 (−91, −51)	−1 (−16, 14)	−11 (−24, 23)
NS-RNFL	−44 (−54, −23)	−47 (−56, −18)	6 (−19, 21)	8 (−12, 19)
Nasal-RNFL	−25 (−40, −10)	−24 (−39, −17)	9 (−5, 18)	3 (−18, 10)
NI-RNFL	−48 (−73, −22)	−54 (−64, 2)	4 (−19, 27)	5 (−21, 17)
TI-RNFL	−96 (−107, −41)	−89 (−111, −40)	9 (−11, 20)	−15 (−45, 15)

**Table 3:** Deviation of optic nerve head retinal nerve fibre layer (RNFL) thickness, with corresponding visual acuity, in LHON and asymptomatic carrier relatives. Visual acuity (VA) is displayed in LogMAR for right eye (RE) and left eye (LE). The RNFL thickness is given in microns, with highlight colour indicating normal thickness (green), above normal (white), borderline thinning (yellow), and thinning (red). The optic nerve segments included are temporal optic nerve (ON-T), temporal superior (ON-TS), nasal superior (ON-NS), nasal (ON-N), nasal inferior (NI) and temporal inferior (TI). C6 left eye is excluded from table as OCT was not available.

	ID	Eye	VA	Global Nerve	ON-T	ON-TS	ON-NS	ON-N	ON-NI	ON-TI
LHON	C1	RE	1.8	−59	−48	−96	−54	−30	−61	−118
		LE	1.5	−63	−45	−90	−66	−40	−68	−114
	C2	RE	2.4	−48	−47	−98	−35	−20	−22	−93
		LE	2.1	−45	−57	−91	−35	−17	2	−88
	C4	RE	2.7	−74	−56	−99	−77	−53	−70	−131
		LE	2.4	−71	−57	−110	−70	−51	−56	−115
	C6	LE	1	−50	−50	−66	−47	−24	−54	−89
	C7	RE	1.8	−64	−48	−69	−52	−56	−94	−99
		LE	1.8	−58	−46	−77	−52	−39	−64	−108
	E1	RE	1	6	13	−6	−28	−2	5	50
		LE	1	11	−2	15	−18	10	34	40
	C9	RE	1.6	−61	−44	−97	−54	−35	−80	−103
		LE	1.9	−62	−47	−95	−56	−36	−78	−111
	C10	RE	1.4	−32	−49	−31	−9	−13	−34	−59
		LE	1.3	−32	−46	−51	2	−24	−30	−40
	E2	RE	1.5	9	−21	36	63	9	−21	12
		LE	1.5	8	−33	34	53	−1	8	33
Asymptomatic	U1	RE	0	2	5	−13	−21	12	3	20
		LE	0.2	0	−3	−19	−32	11	12	23
	U2	RE	0	−39	−47	−61	−12	−12	−38	−90
		LE	0.2	−42	−47	−67	−17	−21	−35	−87
	U3	RE	0.1	−7	10	1	−13	−16	−27	−2
		LE	0.2	−12	0	−13	3	−19	−21	−21
	U4	RE	0.1	12	−6	−8	24	16	60	−1
		LE	0.2	13	−10	22	54	1	50	−8
	U5	RE	0	23	13	1	38	26	42	22
		LE	0	23	8	42	53	15	37	7
	U6	RE	0.1	−9	−12	−23	−22	11	4	−24
		LE	0.1	−11	−4	−23	−1	1	−21	−36
	U7	RE	0.1	4	2	13	9	5	−20	14
		LE	0	7	−1	15	12	10	3	8
	U8	RE	0.1	8	−5	12	3	7	30	17
		LE	2.4	−26	−52	−45	−11	5	15	−70
	U9	RE	0	−1	8	−3	−29	−3	−19	32
		LE	0	2	−5	−1	−14	4	4	32
	U10	RE	0	24	11	30	10	20	62	35
		LE	0	24	5	34	12	22	61	34
	U11	RE	0	−9	−12	−10	20	−10	−16	−30
		LE	0	−10	−9	−14	17	−17	1	−36
	U12	RE	0	17	0	17	14	36	22	8
		LE	0.5	14	3	34	25	16	−30	13
	U13	RE	0	20	−2	38	31	24	26	20
		LE	0	−32	11	25	44	2	23	19
	U14	RE	1.8	−24	−57	−70	24	17	9	−78
		LE	0	−31	−52	−9	15	−24	−36	−70
	U15	RE	0	−5	−2	18	−28	−13	−22	9
		LE	2.1	−40	−48	−36	−27	−23	−37	−80
	U16	RE	0.4	−11	−8	−30	−27	−3	−2	−7
		LE	0	−3	4	−27	−3	7	11	−24

**Table 4:** Optic nerve function including logMAR visual acuity, P100 latency and visual field loss, and mitochondrial mutation, heteroplasmy and haplogroup, and pedigree, in LHON and asymptomatic maternal carrier relatives is shown. The P100 latency is given in milliseconds (ms), with deviation from mean normal value (112 ms) to above 128 ms, or non-recordable (NR), or interocular difference greater than 10 ms highlighted in red as delayed, or if normal is highlighted in green. Amblyopia is reported as present (+) or absent (-).

	ID	Eye	VA	P100 Latency (Deviation)	Visual Field Loss	Amblyopia	Mitochochondrial Mutation Heteroplasmy			Mt Haplogroup	Pedgiree
							3460 G>A	11778 G>A	14484 T>C		
LHON	C1	RE	1.8	NR	3	-		100		H	2
		LE	1.5	NR	3						
	C2	RE	2.4	NR	3	-		100		J	3
		LE	2.1	NR	3						
	C3	RE	0.9	137 (25)	2	-		100		J	4
		LE	0.8	149 (37)	2						
	C4	RE	2.7	NR	4	-		100		HV	5
		LE	2.4	NR	4						
	C5	RE	2.7	193 (81)	4	-		100		HV	5
		LE	2.4	NR	4						
	C6	RE	1	NR	3	-		80		J	6
		LE	1	NR	3						
	C7	RE	1.8	NR	3	-		100		HV	7
		LE	1.8	NR	3						
	E1	RE	1	NR	1	+		100		U	8
		LE	1	NR	1						
	C8	RE	2.1	NR	4	-		100		HV	7
		LE	2.1	NR	4						
	C9	RE	1.6	NR	3	-	100			I	10
		LE	1.9	NR	3						
	C10	RE	1.4	NR	2	-				HV	11
		LE	1.3	NR	2						
	E2	RE	1.5	NR	3	-			100	J	13
		LE	1.5	NR	2						
Unaffected	U1	RE	0	118 (6)	2	+		65		U	1
		LE	0.2	120 (8)	2						
	U2	RE	0	126 (14)	2	-		98		U	1
		LE	0.2	130 (18)	2						
	U3	RE	0.1	112 (0)	2	-		100		H	2
		LE	0.2	111 (−1)	2						
	U4	RE	0.1	107 (−5)	2	-		100		J	3
		LE	0.2	107 (−5)	1						
	U5	RE	0	NA	2	-		100		HV	4
		LE	0	NA	1						
	U6	RE	0.1	114 (2)	1	-		100		J	5
		LE	0.1	117 (5)	1						
	U7	RE	0.1	110 (−2)	1	-		88		J	6
		LE	0	108 (−4)	1						
	U8	RE	0.1	94 (−18)	3	+		100		HV	7
		LE	2.4	121 (9)	1						
	U9	RE	0	107 (−5)	1	-		100		U	8
		LE	0	114 (2)	1						
	U10	RE	0	102 (−10)	1	-		100		HV	7
		LE	0	106 (−6)	1						
	U11	RE	0	100 (−12)	1	-		100		H	9
		LE	0	106 (−6)	1						
	U12	RE	0	98 (−14)	1	-	10			I	10
		LE	0.5	97 (−15)	1						
	U13	RE	0	103 (−9)	1	-				HV	11
		LE	0	106 (−6)	11						
	U14	RE	1.8	112 (0)	3	+		100		H	12
		LE	0	120 (+8)	2						
	U15	RE	0	106 (−6)	2	+		100		H	12
		LE	2.1	125 (13)	3						
	U16	RE	0.4	117 (5)	2	+			100	J	13
		LE	0	111 (−1)	2						

## Data Availability

Data is retained by this research group and may be available on request.
